# A Review of Current and Pipeline Drugs for Treatment of Melanoma

**DOI:** 10.3390/ph17020214

**Published:** 2024-02-07

**Authors:** Nicole Natarelli, Sarah J. Aleman, Isabella M. Mark, Jasmine T. Tran, Sean Kwak, Elizabeth Botto, Shaliz Aflatooni, Michael J. Diaz, Shari R. Lipner

**Affiliations:** 1 Morsani College of Medicine, University of South Florida, Tampa, FL 33602, USA; 2 School of Medicine, Louisiana State University, New Orleans, LA 70112, USA; 3 College of Medicine, University of Florida, Gainesville, FL 32610, USA; 4 School of Medicine, Indiana University, Indianapolis, IN 46202, USA; 5 Department of Dermatology, Weill Cornell Medicine, New York City, NY 10021, USA

**Keywords:** melanoma, targeted therapy, oncolytic virus therapy, combination therapy, immune checkpoint inhibition, transfer therapy

## Abstract

Malignant melanoma is the most aggressive form of skin cancer. Standard treatment options include surgery, radiation therapy, systemic chemotherapy, targeted therapy, and immunotherapy. Combining these modalities often yields better responses. Surgery is suitable for localized cases, sometimes involving lymph node dissection and biopsy, to assess the spread of the disease. Radiation therapy may be sometimes used as a standalone treatment or following surgical excision. Systemic chemotherapy, while having low response rates, is utilized as part of combination treatments or when other methods fail. The development of resistance to systemic chemotherapies and associated side effects have prompted further research and clinical trials for novel approaches. In the case of advanced-stage melanoma, a comprehensive approach may be necessary, incorporating targeted therapies and immunotherapies that demonstrate significant antitumor activity. Targeted therapies, including inhibitors targeting BRAF, MEK, c-KIT, and NRAS, are designed to block the specific molecules responsible for tumor growth. These therapies show promise, particularly in patients with corresponding mutations. Combination therapy, including BRAF and MEK inhibitors, has been evidenced to improve progression-free survival; however, concerns about resistance and cutaneous toxicities highlight the need for close monitoring. Immunotherapies, leveraging tumor-infiltrating lymphocytes and CAR T cells, enhance immune responses. Lifileucel, an FDA-approved tumor-infiltrating lymphocyte therapy, has demonstrated improved response rates in advanced-stage melanoma. Ongoing trials continue to explore the efficacy of CAR T-cell therapy for advanced melanoma. Checkpoint inhibitors targeting CTLA-4 and PD-1 have enhanced outcomes. Emerging IL-2 therapies boost dendritic cells, enhancing anticancer immunity. Oncolytic virus therapy, approved for advanced melanoma, augments treatment efficacy in combination approaches. While immunotherapy has significantly advanced melanoma treatment, its success varies, prompting research into new drugs and factors influencing outcomes. This review provides insights into current melanoma treatments and recent therapeutic advances.

## 1. Introduction

Melanoma is a tumor that forms due to the malignant transformation of melanocytes, pigment-producing cells found predominantly in the skin [1]. Ultraviolet light radiation from sunlight causes the accumulation of genetic mutations and is the main environmental risk factor for melanoma development [2]. Although melanoma represents only approximately 1% of malignant skin tumors, it is the most aggressive and deadliest form of skin cancer due to its metastatic potential [3]. Melanoma incidence has been steadily rising in the United States and worldwide, with an estimated 96,480 adults diagnosed with melanoma in the United States in 2019, and accounting for 5.5% of new cancer cases [4,5].

Melanoma is categorized primarily by tumor, node, and metastasis (TNM) staging in patients with precancer (stage 0), local disease (stage I-II), node-positive disease (stage III), and advanced metastatic disease (stage IV) [6]. Tumor thickness (Breslow depth), lymph node involvement, the extent of ulceration, mitotic rate, and the presence of distant metastasis are used for staging and assessing the risk of recurrence [7]. The five-year relative survival rate for patients with stage 0 melanoma is 97%, compared with approximately 10% for patients with stage IV disease [8], highlighting the importance of continued research in advanced melanoma treatment.

There have been significant advancements in melanoma treatment in the past few decades [1]. Although the incidence of melanoma cases continues to increase, mortality from advanced melanoma has decreased in the past decade given the recent advances in treatment [9]. The known genetic drivers of melanoma include B-raf proto-oncogene (BRAF), neurofibromin 1 (NF1), and NRAS mutations. While standard therapies have traditionally included surgery, radiation therapy, and systemic chemotherapy, the development of targeted therapy and immunotherapy has revolutionized the management of melanoma [10,11,12]. In particular, advanced melanoma treatment often requires a multidisciplinary approach with combination therapies to achieve better responses. Combination approaches using different treatment modalities, such as targeted therapy and immunotherapy, have demonstrated synergistic effects and improved outcomes in select patients [13].

Clinical trials are evaluating pharmacologic agents for the treatment of melanoma, with a particular emphasis on targeted therapy and immunotherapy. The importance of preclinical studies identifying novel therapeutic targets cannot be understated. Current preclinical studies have identified new potential targets for precision melanoma therapy, including CD126, chondroitin sulfate proteoglycan 4 (CSPG4), tandem CD70 and B7-H3, and α_v_β_3_ integrin [14]. Furthermore, novel therapeutic strategies are emerging as promising treatment modalities, including oncolytic virus therapy and the interventional augmentation of immunotherapy efficacy.

Despite the recent advancements in the pharmacologic treatment of advanced melanoma, evaluating and predicting the pharmacologic efficacy in each patient remain challenging. Although immunotherapy continues to revolutionize melanoma treatment, particularly in patients with previously refractory disease, response to immunotherapy remains highly variable among patients and results in long-term survival in about 50% of melanoma patients [15]; therefore, an important area of melanoma immunotherapy research is focused on identifying predictors of immunotherapy response and strategies to augment the efficacy of immunotherapy in refractory patients.

This review highlights the current treatment landscape and recent advances in melanoma treatment, including targeted therapy, immunotherapy, combination approaches, and emerging therapies.

## 2. Current Treatment Landscape for Melanoma

There have been important advancements in the melanoma treatment landscape in the past few decades [1]. Therapeutic options for melanoma are broadly classified into two categories: standard therapies and targeted therapies [16]. While standard therapies have traditionally included surgery, radiation therapy, and systemic chemotherapy, developments in targeted therapies and immunotherapies have revolutionized the management of melanoma [10,11,12]. 

Surgical excision remains the primary treatment for localized melanoma. The tumor is excised with margins between 0.5 cm and 2 cm, depending on the depth of the tumor invasion (in situ: 0.5–1.0 cm margin; ≤1.00 mm depth: 1.0 cm margin; 1.01–2.00 mm depth: 1.0–2.0 cm margin; and ≥2.01 mm depth: 2.0 cm margin) [17]. Surgery offers a high cure rate, particularly for thin, non-invasive tumors. For more advanced cases, lymph node dissection may be necessary. Although surgical intervention can effectively treat localized melanoma, it is not curative for advanced, metastatic disease [16].

Radiation therapy utilizes high-energy X-rays or particles to target melanoma, particularly when localized surgery is not feasible or to reduce recurrence following surgery. The effectiveness of radiation therapy alone is limited in treating metastatic melanoma and is mostly used for palliative purposes and symptomatic relief [18].

Standard systemic chemotherapy for melanoma involves the administration of drugs that kill rapidly dividing cells throughout the body, including cancer cells [19]. However, melanoma has a limited response to conventional chemotherapy, leading to its diminished use in recent years due to the emergence of more effective treatments. Chemotherapy is primarily utilized for advanced melanoma in combination with other treatments or when the melanoma fails to respond to other treatments. 

Despite recent advances, standard therapies are associated with several challenges and limitations. Melanoma may develop resistance to traditional treatments, leading to disease recurrence and progression [20]. This resistance is often attributed to the aggressive nature of melanoma and its ability to adapt to the treatment environment. Overcoming resistance remains a significant challenge in effectively managing advanced melanoma. Standard therapies, such as chemotherapy, can also cause significant side effects due to their non-specific action on healthy cells [19]. Patients may experience fatigue, nausea, hair loss, and other side effects. Reducing treatment-related toxicity while maintaining efficacy is a crucial goal in optimizing melanoma therapy. 

In addition, standard therapies may have limited efficacy against advanced melanoma stages, and are associated with poor outcomes [10]. Therefore, there is important need for more effective treatment approaches to improve survival rates and enhance patient quality of life. Advanced melanoma cases often require a multidisciplinary approach with combination therapies or novel agents to achieve better responses. Combining different treatment modalities, such as targeted therapies and immunotherapies, has synergistic effects and improved outcomes in some patients [13]. Clinical trials exploring various combination regimens are ongoing to identify the most effective treatment approaches for different subsets of melanoma patients.

The treatment landscape for melanoma has evolved significantly, with targeted therapies and immunotherapies revolutionizing patient care. Standard therapies, including surgery, radiation therapy, and chemotherapy are still utilized, but their limitations in advanced melanoma cases necessitate continued research into novel and combination approaches. The challenges posed by standard therapies necessitate the development of novel pharmacologic agents that target specific molecular pathways implicated in melanoma growth and progression. Targeted therapies have emerged as a promising approach that focuses on the unique genetic and molecular characteristics of melanoma tumors [21]. With ongoing advancements and clinical trials, the future holds promise for more effective and personalized treatments for melanoma patients, ultimately improving survival rates and quality of life.

## 3. Targeted Therapies 

Beyond standard therapies, significant progress has been made in understanding the molecular drivers of melanoma, leading to the development of therapies that specifically target aberrant signaling pathways. These therapies offer a personalized approach, inhibiting key molecules responsible for tumor growth and progression. Several targeted agents have been approved for the treatment of melanoma, and ongoing research continues to explore new targets and combination strategies.

Agents targeting BRAF and MEK mutations have shown remarkable efficacy in patients with BRAF-mutated melanoma. Vemurafenib and dabrafenib, both BRAF inhibitors, have excellent response rates and overall survival in BRAF-mutated melanoma patients [20]. Cobimetinib and trametinib, MEK inhibitors, have improved progression-free survival and overall survival in combination with BRAF inhibitors [22]. Additionally, novel therapies targeting other molecular pathways, such as c-KIT and NRAS, are being investigated. Imatinib, a c-KIT inhibitor, has shown promise in patients with c-KIT-mutated melanoma [13]. Encorafenib (BRAF inhibitor) and binimetinib (MEK inhibitor) have demonstrated efficacy in patients with NRAS-mutated melanoma [16].

In the past few years, there have been several studies investigating the safety and efficacy of targeted therapies for melanoma. A 2020 randomized, double-blind, controlled study investigated vemurafenib and cobimetinib combination therapy among 514 patients with BRAF V600-mutated melanoma. The combination therapy demonstrated greater efficacy than either drug given in isolation with a placebo, with an improved progression-free survival of 15.1 months compared to 10.6 months with individual therapies [23]. Similarly, a 2021 review detailing the treatment of advanced melanoma found a PD-1 blockade with BRAF/MEK inhibitor combination therapy demonstrated a 5-year overall survival benefit of 30–40% among melanoma patients [16]. A 2023 systematic review demonstrated that BRAF and MEK inhibitor combination therapy decreased the risk of progression or death compared to monotherapy in women with BRAF-mutant melanoma with a pooled progression-free survival and overall survival hazard ratio of 0.50 (95% CI 0.41–0.61). The combined targeted therapy demonstrated a smaller effect in men, with a progression-free survival and overall survival hazard ratio of 0.63 (95% CI 0.54–0.74) [24]. 

A 2022 murine study found that mice treated with androgen receptor blockade had significantly improved responses to BRAF-MEK-targeted therapy (*p* = 0.018 and *p* = 0.003) [25]. However, a cross-sectional comparative multicenter study utilizing systemic melanoma patient data between 08/2020 and 03/2021 found that patients treated with targeted therapy reported a significantly worse quality of life compared to patients treated with immune checkpoint inhibitors (*p* = 0.02). In addition, patients who received targeted therapy had a 1.9 times greater incidence of adverse events compared to patients treated with immune checkpoint inhibitors (*p* = 0.01), suggesting that potential therapeutic efficacy may be offset by adverse events and reduced quality of life [26]. A summary of these studies is listed in Table 1.

While targeted therapies have improved patient outcomes, challenges, including resistance and toxicities persist. For example, BRAF inhibitors are associated with cutaneous toxicities, including skin rashes and keratoacanthomas [27]. MEK inhibitors are associated with gastrointestinal and ocular side effects [11]. Combination therapies, while effective, can also lead to increased toxicities. Therefore, careful patient selection, monitoring, and the management of adverse events are essential aspects of the clinical use of targeted agents.

Continued research is needed to identify novel therapeutic targets, explore combination strategies, and optimize treatment regimens for patients with melanoma. Overall, the integration of standard therapies, immunotherapies, and targeted agents holds great promise in enhancing the prognosis and quality of life for melanoma patients, moving towards more effective and personalized melanoma management strategies.

## 4. Immunotherapies

Melanoma immunotherapies have revolutionized the landscape of melanoma treatment, offering potential improved patient outcomes. Immunotherapy employs the body’s own immune system to diminish cancer burden by targeting the endogenous immune system rather than the tumor cells, offering an alternative to chemotherapy or radiation approaches with non-specific targets [11,28]. Furthermore, as traditional treatments are often associated with significant adverse effects on normal cells, impacting patient quality of life, emerging advances in immunotherapies are promising for the future of patient care. 

### 4.1. Adoptive Cell Transfer Therapies 

Adoptive Cell Transfer (ACT) Therapies describe the collection and modification of a patient’s immune cells, such as T cells, to amplify their tumor-targeting capability [29]. Modified T cells, including chimeric antigen receptor (CAR) T cells or tumor-infiltrating lymphocytes (TILs), are returned to the patient to provide an enhanced immune response targeting melanoma cells [29]. Emerging investigations regarding the efficacy and optimal utilization of ACTs are largely centered around in vivo models. A pilot study including three patients with distinct melanoma subtypes (mucosal, superficial spreading, and acral) found TIL therapy to be a potentially promising treatment modality for melanoma, with various immunostimulatory and immunosuppressive factors influencing treatment efficacy. Furthermore, adverse effects such as neutropenia were effectively managed with supportive regimens, such as granulocyte colony-stimulating factor (G-CSF) administration [30]. TIL therapies reduce tumor mutational burden and neoantigen loads among patients with metastatic melanoma (*n* = 181) [31]. Factors loosely associated with TIL—tumor heterogeneity and T-cell phenotype—are stipulated to influence the response rates to ACT [31]. One promising pipeline TIL drug is Lifileucel, a one-time cell therapy for metastatic melanoma. An open-label, single-arm study reported an objective response rate of 36.4% with a mean response time of 1.9 months among patients treated with Lifileucel (*n* = 66) [32]. In the context of CAR T-cell therapy, a number of novel strategies have been propositioned to enhance CAR T-cell potency, including optimizing the activation and persistence of CAR T-cells, improving their ability to infiltrate tumor tissues, and overcoming immunosuppressive signals within the tumor microenvironment. Among many tested against melanoma, these include the use of IFNAR1 (Interferon Alpha And Beta Receptor Subunit 1) to augment the combination of inflammatory adjuvants [33], and H9T, an engineered IL-2 partial agonist that promotes the expression of T-cell transcription factor 1 and CAR-modified CD8+T cells, yielding greater tumor infiltration and survival [34]. In sum, ACT therapies represent promising avenues in the treatment of melanoma; however, adoptive cell therapy is less effective for solid tumors due to the poor homing, proliferation, and survival of transferred cells [35]. Strategies to increase effectiveness include combination management strategies and the expression of transgenes to enhance the homing, penetration, and persistence of transferred cells. Beyond the need for greater clinical trial data, future investigations are geared towards refining patient response biomarkers, reducing adverse effects, and improving patient access and affordability.

### 4.2. Immune Checkpoint Inhibitors

Immune checkpoint inhibitors (CPIs) restore and enhance the immune response for recognizing and eliminating cancerous cells [12]. CPIs for melanoma treatment primarily targeted two main checkpoint proteins: cytotoxic T-lymphocyte-associated antigen 4 (CTLA-4) and programmed cell death protein 1 (PD-1). Neoadjuvant immunotherapies with CTLA-4 and PD-1 have demonstrated significant pathological responses with a relapse-free survival among approximately 80% of patients with stage III melanoma [36]. These proteins are implicated in immune response regulation and the prevention of the over-activation of the immune response which could damage healthy tissue [12]. Resistance to the PD-1 blockade is detrimental to cancer treatment as it limits treatment efficacy. Emerging approaches to overcoming the resistance are promising for future treatments. TANK-binding kinase 1 has an established role: coordinating an innate immune response to viruses along with other invading pathogens [37]. In a 2023 study utilizing multiple experimental model systems with genetic and pharmacologic tools, there was a significant effect from the genetic deletion of TBK1, as it sensitizes tumors to immune attacks. This inhibition could overcome the resistance to the PD-blockade [37]. The upregulation of the PD-1 protein was correlated with the indolamine 2,3-dioxygenase 1 and current pharmacological treatments including nivolumab [38,39]. 

### 4.3. Interleukin-2 and Other Cytokines 

IL-2 is a pro-inflammatory and immunoregulatory cytokine that regulates the T cell response. Recently, IL-2 has received significant recognition for its role in the promotion of the induction, survival, and function of CD8+ effector T-cells, with the addition of IL-2 overcoming the resistance to neoadjuvant anti-CTLA4 and anti-PD-1 [36]. Furthermore, IL-2 stimulates dendritic cell (DC) formation through lymphoid cells in both mice and humans, enhancing antitumor immunity [40]. Recent reports of modifications to the IL-2 approach are promising for melanoma treatment. Re-engineered IL-2 therapies have been implicated in longer in vivo half-lives, and they target specific receptor conformations, which reduces toxicity among patients with melanoma [41]. Despite these advances, it is imperative to consider the patient and curate a personalized approach for their care due to the array of potential adverse effects and benefits associated with this treatment method. 

In addition to interleukin-2, there are several other cytokines and chemokines that are associated with melanoma prognosis and progression, including several that have been associated with an improved overall survival. In a 2019 study, the cytokines CCL3, CCL4, IFN-γ, and IL-10 were present at higher levels in melanoma patients compared to the control group. Some of these cytokines were associated with the prognosis and progression of melanoma. Specifically, CCL3 levels increased as tumor growth progressed and had a direct correlation with the presence of ulceration in the primary tumor. Also, IFN-γ and IL-10 were present at higher levels in stage I patients [42]. In another 2019 study analyzing the effect of intratumor immune responses on overall survival, certain immune response-related cytokines and chemokines including IFN-γ and TGFB1 correlated with a favorable overall survival [43]. In a 2023 study, melanoma patients with a higher expression of TNFSF13, CXCL10, and CXCL13 exhibited higher infiltration by core tertiary lymphoid structure cell populations. The presence of these cytokines was associated with an improved survival rate and a pro-inflammatory tumor microenvironment that supports lymphoid aggregates and tertiary lymphoid structures [44]. The association of certain cytokines with an improved overall survival in melanoma patients in consideration of a patient’s unique tumor microenvironment as well as the further exploration of immunologic signaling pathways as a source of potential therapeutic insights guide future treatment developments. 

### 4.4. Oncolytic Virus Therapy

Oncolytic virus (OV) therapy utilizes modified viruses to selectively target, infect, and induce immunogenic cancer cell death while sparing normal healthy cells [33,45]. Thus far, OV Pexa-Vec (Pexastimogene Devacirepvec) has been the most well studied [45]. Pexa-Vec has shown significant efficacy as a single agent among both low- and high-dose injections among patients with hepatocellular carcinoma. A 2022 study reported treatment toleration among patients with metastatic melanoma with maintained clinical efficacy [45]. The application of OV enhances CAR T cell efficacy via enhanced proliferation, CAR-directed antitumor function, and memory, leading to prolonged survival in mouse models with subcutaneous melanoma [46]. High levels of Fas and PD1 were observed on CAR T-cells within an infected tumor; the expression of respective ligands may be regulated by OV [33]. OV theoretically may be used as a platform for combination therapies to treat tumors that are unresponsive to CPI therapies [47]. However, the immunological heat generated from tumor infection from an OV is a complex multifaceted process, potentially decreasing the optimization of OVs as a form of immunotherapy; therefore, it is imperative to consider the condition of the patient and optimal treatment options prior to implementing this approach [33]. 

While Pexa-Vec is the most well studied, additional oncolytic viruses have been evaluated for the management of melanoma. Talimogene laherparecpvec (T-VEC) is an oncolytic virus that is approved for patients with stage IIIB, IIIC, or IV melanoma. Following intratumoral injection, the virus initiates local and systemic immunological responses that contribute to tumor cell lysis. Furthermore, the virus triggers the release of tumor-derived antigens, which are important for the subsequent activation of tumor-specific effector T-cells [48]; however, T-VEC administration does not increase overall survival in isolation but instead augments treatment efficacy in combination approaches [49]. Various other oncolytic viruses are in development or are under current clinical investigation, including ONCOS-102 and coxsackievirus A21. 

ONCOS-102 is a modified adenovirus expressing granulocyte–macrophage colony-stimulating factor (GM-CSF) which binds to the desmoglein 2 receptor commonly expressed on tumor cells [49]. Animal and human studies demonstrated the recruitment of natural killer and cytotoxic T cells into the tumor environment following injection. A recently published 2023 study found that treatment with intratumoral ONCOS-102 virotherapy resulted in an objective response rate of 35% following ONCOS-102 and pembrolizumab combination therapy among 21 patients previously refractory to a PD-1 blockade [50]. 

Coxsackievirus A21, an RNA virus that targets intercellular adhesion molecule-1 (ICAM-1) was studied for the treatment of advanced melanoma in combination with immunotherapy [51]. A 2022 study reported an objective response rate of 30% following the treatment of fifty patients treated with intratumoral V937 injections and intravenous ipilimumab. An objective response rate of 47% was observed among anti-PD-1-naïve patients and 21% among those previously refractory to anti-PD-1. Numerous clinical trials are currently investigating other oncolytic viral interventions, including recombinant oncolytic HSV-2 (NCT05070221, NCT04616443), spontaneously attenuated mutant HSV-1 (NCT03153085), and genetically modified HSV-1 (NCT03767348). 

Table 2 summarizes various immunotherapies, their mechanisms of action, and their adverse effects. 

## 5. Combination Approaches 

The complexity of melanoma tumors along with the intricate relationship between the immune system and cancer cells has necessitated the development of a synergistic treatment that may target multiple pathways and mechanisms. While surgical resection is a common component to treatment approaches, immunotherapies including ipilimumab and nivolumab are effective for some unresectable cancers [54]. In addition, this approach has shown promise in combination with other therapy. As of 2023, the utilization of the mitogen-activated protein kinase (MAPK) pathway with CPI—specifically the PD-1 inhibitor—improved antitumor immunity in metastatic melanoma treatment [55]. Combinations of newly FDA-approved therapies targeting PD-1 and LAG-3 via nivolumab and relatlimab, respectively, have shown improved progression-free survival compared to nivolumab monotherapies among patients with advanced melanoma (*n* = 30) [56]. 

The timing of combination approaches (neoadjuvant vs. adjuvant therapy) plays a significant role in patient outcome. In a phase 2 trial, patients with resectable stage IIIB-IVC melanoma that received neoadjuvant and adjuvant treatment with pembrolizumab (*n* = 154) had a longer event-free survival compared to those receiving an adjuvant-only treatment (*n* = 159) (*p* = 0.004), with 72% of neoadjuvant–adjuvant patients having an event-free survival of 2 years compared to 49% among adjuvant-only patients [57]. The rate of grade 3+ adverse effects was similar between patients receiving the neoadjuvant–adjuvant treatment (12%) and those receiving an adjuvant-only treatment (14%) [57].

Multispecific antibodies are another type of therapy that has been heavily discussed as a potential treatment for melanoma. Multispecific antibodies are created by combining different antibody components together to offer overall improved functionality. These antibodies may potentially disrupt multiple tumor-associated antigens and enhance immune cell activation, leading to higher response rates and delayed resistance development [58]. Multispecific antibodies may target different epitopes on two or more checkpoint molecules and can also crosslink antigens on neighboring cells leading to cell–cell bridging that can achieve greater T cell activation and/or cancer cell killing. In a 2023 study, multispecific antibodies were created against PD-L1, TIGIT, and LAG-3 and efficiently promoted T cell activation and cancer cell killing, and suppressed tumor growth. In the same study, it was proposed that these single-molecule multispecific antibodies have high potential to simplify clinical development and are likely more efficacious in clinical settings than combinations of monospecific checkpoint inhibitors. Multispecific antibodies may have superior efficacy to antibody combinations, including nivolumab/relatlimab [59].

Table 3 summarizes the benefits and challenges of treatment modalities for melanoma. 

## 6. Emerging Therapies and Future Directions

A multitude of registered clinical trials are currently evaluating pharmacologic agents for the treatment of melanoma, including targeted therapies and immunotherapies. Preclinical studies have identified various potential future targets for targeted melanoma therapy, including CD126, chondroitin sulfate proteoglycan 4 (CSPG4), tandem CD70 and B7-H3, and α_v_β_3_ integrin [14]. In addition, novel pharmacologic strategies are under evaluation, including oncolytic virus therapy and the interventional augmentation of immunotherapy efficacy. 

Although immunotherapy has revolutionized the treatment of melanoma, with promising efficacy for previously refractory disease, it is limited by its outcome variability between patients. For largely unknown reasons, immunotherapy achieves long-term survival in only about 50% of melanoma patients [15]. Thus, while novel pharmacologic agents are under investigation, a great portion of pharmacologic melanoma research is currently focusing on identifying predictors of immunotherapy success and strategies to augment immunotherapy efficacy in refractory patients.

For example, the gastrointestinal microbiome composition has recently drawn attention as a predictive biomarker of immunotherapy outcomes [60]. As such, recent studies have investigated microbiota-modulating interventions, such as fecal microbiota transfer, in combination with immunotherapy among advanced melanoma patients. In 2021, there were clinical responses in three of ten patients previously refractory to anti-PD-1 treatment with fecal microbiota transplantation and the reinduction of immunotherapy [61]. Similarly, a 2021 study found clinical benefits in six of fifteen previously refractory patients treated with fecal microbiota transplantation and anti-PD-1 immunotherapy [62]. Additional clinical trials are currently assessing fecal microbiota transfer among advanced melanoma patients (NCT04577729, NCT05251389, and NCT04988841).

Future studies could seek to identify additional predictors of immunotherapy efficacy and assess interventions that modify such predictors in combination with approved immunotherapy. In addition, studies could seek to identify additional targets that may provide therapeutic strategies for targeted therapy.

## 7. Limitations

Despite great advancements in the pharmacologic treatment for advanced melanoma, there are many challenges in evaluating and comparing pharmacologic agents. For example, tumor heterogeneity, both genetically and phenotypically, increases the difficulty in evaluating pharmacological agents that can target various subtypes of melanoma. In addition, studies evaluating therapeutic efficacy may include patients with distinct genetic or phenotypic tumor characteristics. Similarly, studies may include patients who are refractory or naïve to previous treatments, which may suggest differing tumor or patient characteristics. Patients with different stages of the disease may be included, which may result in objective response rates that are not comparable to other studies. Furthermore, trials have differing lengths of long-term follow-up, with shorter studies unable to capture data regarding long-term safety and the development of resistance to the assessed agent over time. Lastly, as the cost and access of various drugs differ, studies evaluating the cost-efficacy of various agents are warranted.

## 8. Conclusions

Melanoma’s high metastatic potential and challenging prognosis in advanced stages necessitate innovative therapeutic approaches, as standard treatments have limitations. Surgical excision is the most effective for early-stage, localized melanoma, while radiation therapy alone is not curative. Chemotherapy resistance may lead to recurrence and progression, and is associated with significant side effects. Understanding these constraints is crucial for enhancing patient outcomes.

Recent breakthroughs in melanoma treatment involve the development of more targeted therapies and immunotherapies. Personalized targeted therapies tailored to individual mutations show promise, and combining them may significantly improve progression-free survival, albeit with increased toxicity. Immunotherapies also demonstrate the recent advances in the field of melanoma treatments. Immune checkpoint inhibitors enhance the immune response and assist in the elimination of cancerous cells. Combining these inhibitors enhances progression-free survival and antitumor responses. Oncolytic virus therapy is effective for many melanoma patients, particularly when used in conjunction with other treatments. Oncolytic virus therapy, especially when combined with other treatments, is effective in some patients. Adoptive cell transfer, including TIL and CAR-T therapies, offers innovative avenues. Despite the significant success of immunotherapy, it is important to note that not all melanoma patients respond equally. Ongoing research aims to understand why responses vary and to develop strategies to enhance the efficacy of immunotherapy. Combining immunotherapy with other treatment modalities, such as targeted therapies, is also an area of active investigation.

Looking forward, strategies to refine drug delivery, integrate biologics and gene therapies, and overcome drug resistance may drive short-term progress in melanoma drug development. We also anticipate greater emphasis on equitable and personalized medicine selection. These changes will likely come at the hands of increased interdisciplinary collaboration, advancements in genomic profiling and biomarker identification, and improvements in survivorship care plans and mental health support.

## Figures and Tables

**Table 1:** Summary of recent advances: targeted therapies.

Author (Year)	Major Results
Gutzmer et al., (2020) [23]	Combination therapy with vemurafenib and cobimetinib was superior to individual therapies in patients with BRAF V600-mutated melanoma, offering an improved progression-free survival of 15.1 months compared to 10.6 months with individual therapies.
Jenkins et al., (2021) [16]	A single-agent PD-1 blockade along with BRAF/MEK inhibitor combination therapy demonstrated a 5-year overall survival benefit of 30–40%.
Vellano et al., (2022) [25]	In a murine model of melanoma, mice treated with an androgen receptor blockade had significantly improved responses to BRAF-MEK-targeted therapy (*p* = 0.018 and *p* = 0.003).
Pala et al., (2023) [24]	BRAF and MEK inhibitor combination therapy reduced the risk of progression or death in women compared to monotherapy, with a progression-free survival and overall survival hazard ratio of 0.50 (95% CI 0.41–0.61). In men, the hazard ratio was 0.63 (95% CI 0.54–0.74). The study suggests a hormonal influence on the signaling pathways involved in BRAF and MEK inhibition.
Thiem et al., (2023) [26]	Targeted therapy patients reported a significant drop in quality of life compared to immune checkpoint inhibitor patients (*p* = 0.02). Adverse events were 1.9 times higher for patients on targeted therapy (*p* = 0.01), suggesting a decrease in quality of life and a higher risk of adverse events compared to immunotherapy.

**Table 2:** Immunotherapy: mechanism of action and adverse effects [52,53].

Treatment	How They Work	Categories	Examples	Adverse Effects
Adoptive Cell Transfer Therapies (ACT)	Collection and modification of a patient’s immune cells to amplify their tumor-targeting capability	CAR T cells, Tumor-infiltrating lymphocytes (TILs)	Lifileucel	Thrombocytopenia, anemia, febrile neutropenia
Immune Checkpoint Inhibitors (CPI)	Restoration and enhancement of immune response for recognizing and eliminating cancerous cells	CTLA-4 PD-1	Ipilimumab, Penbrolizumab	Fatigue, diarrhea, itching, rash; Fatigue, cough, nausea, rash, itching, joint pain
Interleukin 2 (IL-2)	Activation of the cytotoxic function of natural killer (NK) cells, T lymphocytes, and monocytes		Aldesleukin	Chills, fever, fatigue, nausea, vomiting, diarrhea
Oncologic Virus Therapy (OV)	Use of viruses to target, infect, and kill cancer cells		Talimogene laherparepvec (T-VEC)	Flu-like symptoms, fatigue, chills, nausea

**Table 3:** Benefits and challenges of treatment modalities for melanoma.

	Benefits	Challenges
Surgical Excision	-High cure rate for thin, non-invasive tumors	-May require lymph node dissection for more advanced cases -Not curative for metastatic disease
Radiation Therapy	-May be used when localized surgery is not feasible -Can foster reduced recurrence following surgery	-Limited efficacy in metastatic melanoma -Mostly utilized for palliative purposes and symptomatic relief
Systemic Chemotherapy	-Can be used in metastatic disease	-Melanoma can develop resistance, leading to disease recurrence and progression -Side effects due to non-specific action on healthy cells, including fatigue, nausea, and hair loss
Targeted Therapy	-Targets specific mutations or molecules, reducing systemic side effects	-Melanoma can develop resistance, leading to disease recurrence and progression -BRAF inhibitors are associated with cutaneous toxicities, such as skin rashes and keratocanthomas -MEK inhibitors are associated with gastrointestinal and ocular side effects
Immunotherapy	-Efficacy has been demonstrated in disease cases resistant to other treatment modalities -Augments immune response	-Adoptive cell transfer is less effective for solid tumors due to poor homing, proliferation, and survival of transferred cells -Resistance to immune checkpoint inhibitor blockade can develop -Does not work equitably in all patients, requiring further delineation of patient and tumor characteristics that predict efficacy -Access and affordability

## Data Availability

Data sharing is not applicable.

## Abbreviations

ACT	Adoptive Cell Transfer
CAR	Chimeric Antigen Receptor
CPI	Immune Checkpoint Inhibitors
CTLA	Cytotoxic T-Lymphocyte-Associated Antigen 4
CSPG4	Chondroitin Sulfate Proteoglycan 4
DC	Dendritic Cells
G-CSF	Granulocyte Colony-stimulating Factor
GM-CSF	Granulocyte–Macrophage Colony-Stimulating Factor
ICAM-1	Intercellular Adhesion Molecule-1
IL-2	Interleukin-2
OV	Oncolytic Virus
PD-1	Programmed Cell Death Protein 1
TIL	Tumor-infiltrating Lymphocytes
T-VEC	Talimogene Laherparecpvec
